# Cytotoxicity and Wound Closure Evaluation in Skin Cell Lines after Treatment with Common Antiseptics for Clinical Use

**DOI:** 10.3390/cells11091395

**Published:** 2022-04-20

**Authors:** Laura Ortega-Llamas, María I. Quiñones-Vico, Marta García-Valdivia, Ana Fernández-González, Ana Ubago-Rodríguez, Raquel Sanabria-de la Torre, Salvador Arias-Santiago

**Affiliations:** 1 Cell Production and Tissue Engineering Unit, Virgen de las Nieves University Hospital, 18014 Granada, Spain; laura.ortega.llamas@gmail.com (L.O.-L.); maribelqv@ugr.es (M.I.Q.-V.); martagv9495@gmail.com (M.G.-V.); aur@ugr.es (A.U.-R.); raquelsanabriadlt@gmail.com (R.S.-d.l.T.); salvadorarias@ugr.es (S.A.-S.); 2 Biosanitary Institute of Granada (ibs.GRANADA), 18014 Granada, Spain; 3 Andalusian Network of Design and Translation of Advanced Therapies, 41092 Seville, Spain; 4 Dermatology Department, School of Medicine, University of Granada, 18014 Granada, Spain; 5 Dermatology Department, Virgen de las Nieves University Hospital, 18014 Granada, Spain

**Keywords:** antiseptics, cell migration, cytotoxicity, fibroblasts, keratinocytes, wound healing, wound regeneration

## Abstract

In recent years, new therapies, such as skin cell lines injections, have emerged to promote re-epithelialization of damaged areas such as chronic ulcers or to treat patients with severe burns. Antiseptics are commonly used during wound clinical management to avoid serious infections, but they may delay the healing process due to their apparent cytotoxicity to skin cells. The cytotoxicity of ethanol, chlorhexidine digluconate, sodium hypochlorite, povidone iodine and polyhexanide was evaluated in this in vitro study on human fibroblasts and keratinocytes. Treatments were applied to each cell type culture every 48 h for 14 days. To determine the cytotoxic of antiseptics, cell viability (Live/Dead^®^) and cell proliferation (AlamarBlue™) assays were performed on cell monolayers. Cell migration capacity was evaluated with a wound closure assay. Results showed how chlorhexidine digluconate and ethanol significantly reduced the viability of keratinocytes and inhibited cell migration. Povidone iodine followed by chlorhexidine digluconate significantly reduced fibroblast cell viability. Povidone iodine also inhibited cell migration. Sodium hypochlorite was the least detrimental to both cell types. If epithelial integrity is affected, the wound healing process may be altered, so the information gathered in this study may be useful in selecting the least aggressive antiseptic after treatment with new emerging therapies.

## 1. Introduction

The skin is the first physical protective barrier in the human body against water, microorganisms, mechanical trauma, chemical substances and damage caused by ultraviolet light. It is composed of three layers: the epidermis, dermis and hypodermis [1,2].

In the epidermis, there are four types of cells that form a stratified epithelium. Keratinocytes are the most frequent, and, other less abundant cell types are found among them: melanocytes, Langerhans cells and Merkel cells. The dermis is the layer beneath the epidermis. It consists mainly of connective tissue, fibroblasts and collagen fibers. The hypodermis is the deepest layer, located below the dermis and mainly composed of adipocytes [1,2].

During the regeneration of damaged skin, processes such as cell proliferation, cell migration, secretion of growth factors and specific cytokines, among others, are present. An alteration in any of these events can delay the wound healing process and lead to a chronic wound, which may be the focus of significant bacterial colonization [3]. Burns or hard-to-heal ulcers promote bacterial colonization and biofilm formation. These infections can cause sepsis with fatal consequences for the patient. Therefore, antimicrobial treatments with antibiotics or antiseptics are often required for successful wound healing [4,5]. The correct proliferation of fibroblasts and keratinocytes during the skin regeneration process is essential. Moreover, these cells are mainly involved in the inflammatory response, one of the most crucial phases for the epithelial regeneration process [3].

In order to prevent and treat localized skin infections in clinic, the use of antiseptics has been encouraged due to the emergence of bacterial resistance to antibiotics. Antiseptics are clinically applied topically on the skin to prevent infection due to their ability to reduce microorganisms [6]. Applying antiseptics at low concentrations is recommended since, according to previous studies, high concentrations can damage the treated tissue and affect the cell viability and migration capacity of fibroblasts and keratinocytes, which can delay the healing process [6,7]. Despite their clinical use for wound healing, antiseptics and antibiotics can affect the viability of skin cells, but there are few studies analyzing the impact of antiseptics on fibroblasts and keratinocytes cultured in vitro [8,9].

The main objective of this study was to evaluate the cytotoxicity of common antiseptics for clinical use on fibroblasts and keratinocytes involved in epithelial regeneration in concentrations ranging from the concentration used in clinical practice to a 1% dilution of the stock solution. In addition, it provides information about the effect of these antiseptics on two essential aspects of the wound healing process: cell migration and proliferation. This research provides useful evidence for selecting an adequate antiseptic treatment during wound management where cell suspension or BASS are used as advanced therapy.

## 2. Materials and Methods

### 2.1. Cell Isolation and Culture

Human fibroblasts (HFs) were isolated from skin samples (9 cm^2^) from plastic, dermatological or urological surgery with the prior consent of the patients in compliance with the requirements for human cell and tissue donation (Royal Decree-Law 9/2014, of 4 July) [9].

The dermis and epidermis were separated by mechanical processing. The dermis was incubated for 18–24 h in a 2 mg/mL solution of type I collagenase (Gibco, Thermo Fisher Scientific, Carlsbad, CA, USA). After the incubation time, the dermis was neutralized with a specific medium for dermal fibroblasts (DFM).

Cell suspensions were centrifuged at 1000 rpm for 10 min. Türk (Sigma Aldrich, St. Louis, MO, USA) and Trypan Blue (Sigma Aldrich, St. Louis, MO, USA) solutions were used for cell counting and determining viability after initial processing. Fibroblasts were seeded at a density of 100,000–140,000 cells/cm^2^ at initial processing and at 5000–7000 cells/cm^2^ after passing.

The immortalized human keratinocyte cell line HaCaT was used as a model to study of keratinocyte cytotoxicity [10]. HaCaT cells were seeded at a density of 10,000 cells/cm^2^.

### 2.2. Cell Viability Assays with Antiseptics

HFs and HaCaT cells were seeded in 24-well plates (Thermo Fisher Scientific, Carlsbad, CA, USA) at a density of 10,000 cells/cm^2^. Cell culture monolayers in 24-well plates were treated with five different antiseptics which were applied for three minutes every 48 h for 14 days. For this purpose, the medium was removed before each treatment. Subsequently, 500 µL of the antiseptic solution was applied to each well. After three minutes, the wells were washed with Dulbecco’s phosphate-buffered saline solution (DPBS, Sigma Aldrich, St. Louis, MO, USA) and finally the medium was added to the treated cell wells and incubated at 37 °C, 5% CO_2_ until the next treatments.

The antiseptics used were: 70% ethanol (Betamadrileño SL, Madrid, Spain), 2% chlorhexidine digluconate (HiBiSCRUB**^®^**, Molnlycke Health Care AB, Madrid, Spain), 0.02% sodium hypochlorite (Microdacyn, Sonoma Pharmaceuticals, CA, USA), povidone iodine 100 mg/mL (LAINCO, SA, Barcelona, Spain) and 0.1% polyhexanide (Prontosan, B Braun Medical, Barcelona, Spain), all approved for clinical use.

A range of concentrations of the stock solution of each antiseptic was used: 1%, 5%, 10%, 50% and 100% (concentration used in the clinic). All solutions were diluted in DPBS.

A preliminary study was used to determine the concentration which provided considerable cell viability and showed the most significant difference between different treatments. Finally, the 1% concentration of the stock antiseptic solution (0.7% ethanol, 0.02% chlorhexidine digluconate, 0.0002% sodium hypochlorite, 1 mg/mL povidone iodine and 0.001% polyhexanide) was tested three times and statistical analyses were performed with this concentration.

Cell viability and proliferation were determined at days 3, 7, 10 and 14 using two different protocols: Live/Dead**^®^** Cell Viability Assay (Thermo Fisher Scientific, Carlsbad, CA, USA) and AlamarBlue assay (Invitrogen™ alamarBlue™ HS Cell Viability Reagent, Thermo Fisher Scientific, Waltham, MA, USA).

#### 2.2.1. Live/Dead^®^ Cell Viability Assay

The Live/Dead**^®^** cell viability assay is a colorimetric assay which consists of preparing a staining solution that combines two fluorescent reagents, calcein AM (green fluorescence, Ex/Em 494/517 nm) and ethidium homodimer-1 (red fluorescence, Ex/Em 517/617 nm). It allows live cells to be differentiated from dead cells by staining them green and red, respectively.

The Live/Dead**^®^** staining solution was applied on days 3, 7, 10 and 14 of treatment. Then, it was incubated in the dark for 30 min at room temperature. After the incubation time, the solution was removed, the plates were washed with DPBS, and fluorescence was measured at 405 nm using a Leica DM2000 microscope (Leica, Wetzlar, Germany). The images obtained were analyzed using ImageJ v1.47 software (National Institutes of Health, Bethesda, MD, USA) to determine the percentage of cell viability.

#### 2.2.2. AlamarBlue Cell Proliferation Assay

AlamarBlue™ HS Cell Viability Reagent is a ready-to-use resazurin-based solution which allows proliferation to be quantified from the reducing capacity of living cells [11].

On days 3, 7, 10 and 14 of treatment, alamarBlue reagent was added to each well (10% vol/vol), incubated in the dark at 37 °C, 5% CO_2_ for four hours and fluorescence was measured at 560/590 nm using a 96-well plate [11]. Cell proliferation was measured by the degree of reduction in the reagent and the concentration of live cells (cells/cm^2^) after each treatment, by establishing a standard curve. Measurements at 1% concentration of stock solutions of the antiseptics were tested three times.

### 2.3. Wound Closure Assay

A wound closure assay, also known as a Scratch Test, was performed to determine the impact of antiseptics on cell migration and wound closure.

HFs and HaCaT cells were seeded in 12-well plates (Thermo Fisher Scientific, Carlsbad, CA, USA) at a density of 10,000 cells/cm^2^. When the cells were confluent and adherent to the plate, the DFM was removed and each well was scratched with a sterile 10 µL pipette tip to simulate wound formation [12]. Cells were then treated for three minutes with the different antiseptics mentioned above, at a concentration of 1% of the clinically used stock solution. Control cells were left untreated. Cell debris and traces of the antiseptic solution were removed by washing with DPBS [12]. Then, the cells were incubated and images were taken of the scraped area of each well until the control was completely closed.

The images were analyzed with ImageJ software. Equations (1) and (2) were used to calculate the percentage of wound closure and cell migration rate (µm/h), respectively [13]. The percentage of wound closure at hour 0 was considered 0% and the percentage of reduction of the scratched area was calculated at 6, 12 and 24 h for HFs and at 12, 24, 36 and 48 h for HaCaT cells. Wound closure was monitored until the control was completely closed in each cell line [12,13].

(1) $$Wound\;closure\;\left(\%\right)=\left(\frac{A_{t=0}-A_{t}}{A_{t=0}}\right)\times 100\;$$

(2) $$Cell\;migration\;rate\;\left(\frac{\mu m}{h}\right)=\left(\frac{W_{i}-W_{f}}{t}\right)$$

where “$A_{t=0}$” is the area of the initial wound just after scratching, “$A_{t}$” is the wound area after “n” hours of initial scratching, “*W_i_*” is the average of the initial wound width in µm, “*W_f_*” is the average of the final wound width in µm and “*t*” is the time of the assay (in hours) until the control wound closed [12].

### 2.4. Statistical Analysis

Statistical analysis of the data was carried out using the program GraphPad Prism (GraphPad Prism 8.0 Software, Inc., La Jolla, CA, USA). The data obtained are expressed as the mean ± the standard error of the mean (SEM). For the analysis, a factorial analysis of variance (ANOVA) was applied to determine the effect of each factor present. Once the ANOVA test was applied, a post hoc analysis was performed with the Tukey’s test for all factors to determine the degree of significance when comparing the factor classes. *p* ≤ 0.05 was considered statistically significant.

## 3. Results

### 3.1. High Concentrations of Antiseptics Cause a Total Reduction of Cell Viability in Skin Cell Lines

All treatments caused high toxicity in both HaCaT cells and HFs when the concentrations used clinically were applied (70% ethanol, 2% chlorhexidine digluconate, 0.02% sodium hypochlorite, povidone iodine 100 mg/mL and 0.1% polyhexanide). At day 3, treatments with these concentrations caused 100% cell death compared to the untreated control. When the concentration of the stock solutions for the antiseptics tested was reduced to 50%, 10% or 5%, the impact on cell viability remained highly noticeable in all treatments. A significant reduction in cell viability was observed when comparing each treatment with the untreated control. However, after treatment with sodium hypochlorite, a smaller reduction in cell viability was observed. Sodium hypochlorite was found to be the least toxic antiseptic on skin cell lines at the concentrations tested (Figures S1–S10).

### 3.2. Common Antiseptics for Clinical Use Diluted to 1% Affect Cell Viability in Skin Cell Lines

#### 3.2.1. Chlorhexidine Digluconate and Ethanol Affected the Viability of HaCaT Cells More Than the Other Treatments

**Figure 1:**
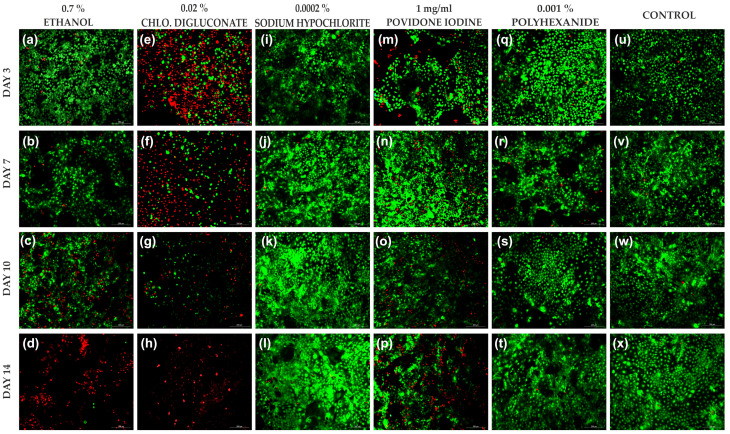
LIVE/DEAD^®^ images of HaCaT cells after each treatment at 1% of stock solution and the control at days 3, 7, 10 and 14. (**a**–**d**) HaCaT cells after ethanol (0.7%) treatment at days 3, 7, 10 and 14, respectively. (**e**–**h**) HaCaT cells after chlorhexidine digluconate (0.02%) treatment at day 3, 7, 10 and 14, respectively. (**i**–**l**) HaCaT cells after sodium hypochlorite (0.0002%) treatment at days 3, 7, 10 and 14, respectively. (**m**–**p**) HaCaT cells after povidone iodine (1 mg/mL) treatment at days 3, 7, 10 and 14, respectively. (**q**–**t**) HaCaT cells after polyhexanide (0.001%) treatment on days 3, 7, 10 and 14, respectively. (**u**–**x**) Control (without treatment) at days 3, 7, 10 and 14, respectively. Dead cells are represented in red and live cells in green. *n* = 3. Magnification 10×.

The concentration range tested was reduced to 1% of the stock solutions. At this concentration, treatments with chlorhexidine digluconate and ethanol in HaCaT cells significantly reduced the percentage of living cells compared to the other treatments, turning out to be the most toxic antiseptics for this cell line. However, polyhexanide and sodium hypochlorite reflected similar cell viability to the untreated control (Figure 1 and Table S1).

**Figure 2:**
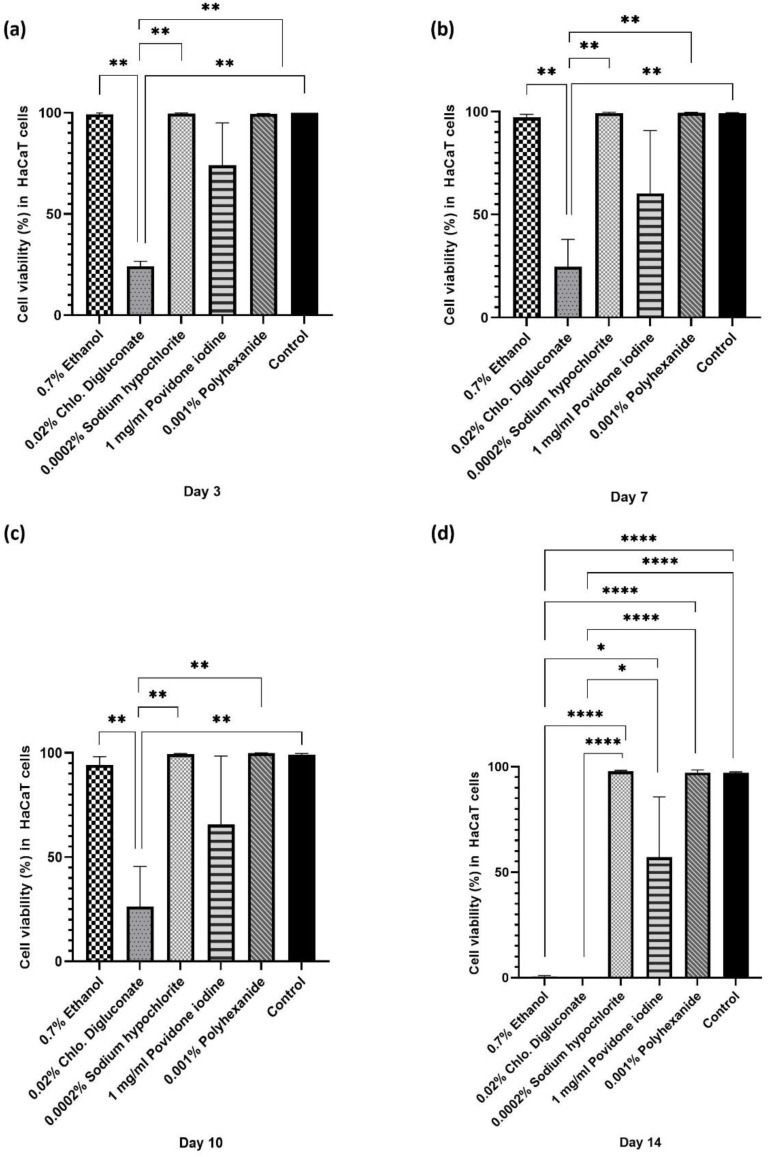
Statistical analysis of cell viability in HaCaT cells after antiseptic treatment on days 3, 7, 10 and 14. Bar graph of cell viability percentage in HaCaT cells at (**a**) day 3, (**b**) day 7, (**c**) day 10, and (**d**) day 14 of treatment. * *p* ≤ 0.05; ** *p* ≤ 0.01; **** *p* ≤ 0.0001. *n* = 3. *p* values ≤ 0.05 were considered statistically significant.

Significant differences were observed between the different antiseptics and days of treatment (Figure 2). At day 3, 7 and 10, significant differences were observed in cell viability after treatment with chlorhexidine digluconate compared to ethanol, sodium hypochlorite, polyhexanide and the control (Figure 2a–c). At day 14, a significant reduction in cell viability was observed after treatment with chlorhexidine digluconate and ethanol compared to sodium hypochlorite, polyhexanide, povidone iodine and the control (Figure 2d).

#### 3.2.2. Povidone Iodine and Chlorhexidine Digluconate Reduced HFs Cell Viability Compared to the Other Treatments

**Figure 3:**
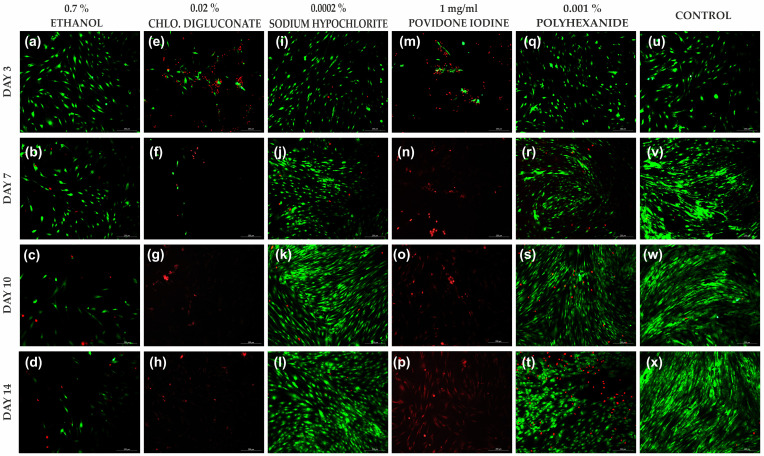
LIVE/DEAD^®^ images of HFs after each treatment with 1% antiseptic stock solution and the control at days 3, 7, 10 and 14. (**a**–**d**) HFs after ethanol (0.7%) treatment at days 3, 7, 10 and 14, respectively. (**e**–**h**) HFs after chlorhexidine digluconate (0.02%) treatment at days 3, 7, 10 and 14, respectively. (**i**–**l**) HFs after sodium hypochlorite (0.0002%) treatment at days 3, 7, 10 and 14, respectively. (**m**–**p**) HFs after povidone iodine (1 mg/mL) treatment at days 3, 7, 10 and 14, respectively. (**q**–**t**) HFs after polyhexanide (0.001%) treatment at days 3, 7, 10 and 14, respectively. (**u**–**x**). Control at days 3, 7, 10 and 14, respectively. Dead cells are represented in red and live cells in green. *n* =3. Magnification 10×.

In HFs at 1% concentration of antiseptics in the stock solution, chlorhexidine digluconate, ethanol and povidone iodine significantly reduced the percentage of live cells compared to the other treatments. Povidone iodine and chlorhexidine digluconate were the antiseptics that caused the greatest impact. However, treatment with sodium hypochlorite reflected a cell viability similar to the untreated control (Figure 3 and Table S2).

**Figure 4:**
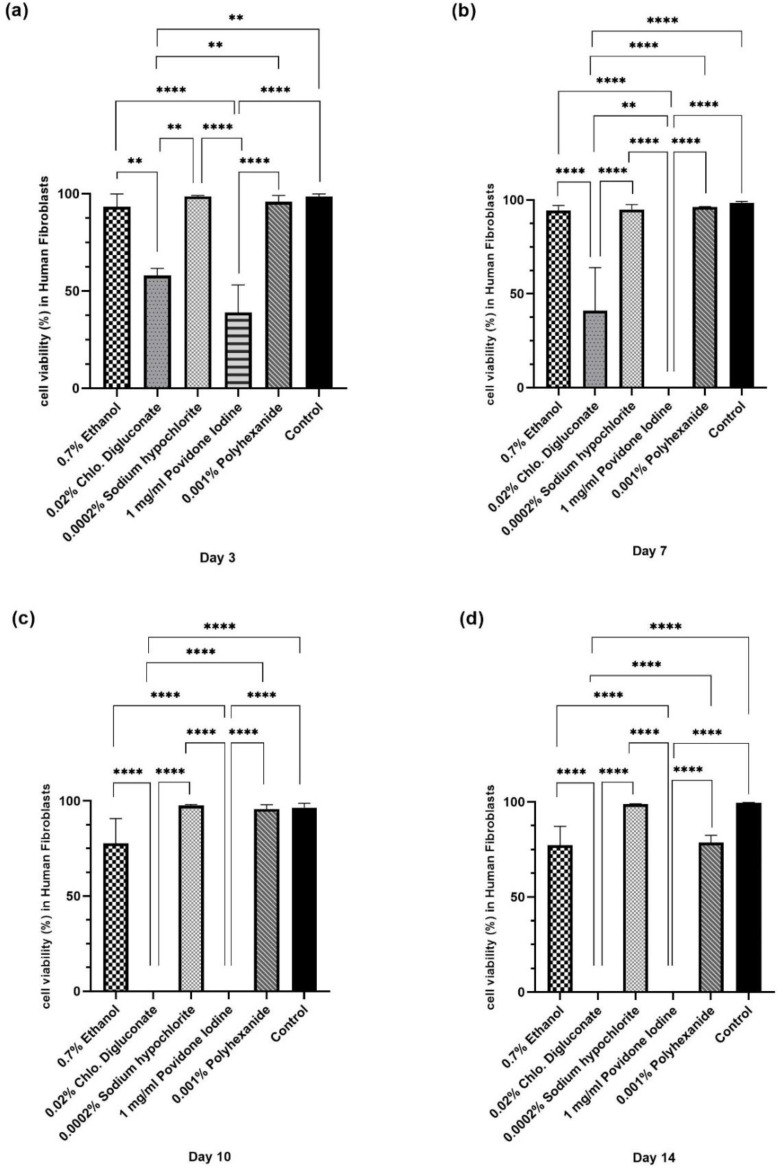
Statistical analysis of cell viability in HFs after antiseptic treatment on days 3, 7, 10 and 14. Bar graph of cell viability percentage in HFs at (**a**) day 3, (**b**) day 7, (**c**) day 10, and (**d**) day 14 of treatment. ** *p* ≤ 0.01; **** *p* ≤ 0.0001. *n* = 3. *p* values ≤ 0.05 were considered statistically significant.

At day 3, cell viability was significantly reduced after treatment with chlorhexidine digluconate and povidone iodine, compared to ethanol, sodium hypochlorite, polyhexanide and the control (Figure 4a). At day 7, a significant decrease in cell viability was seen after treatment with povidone iodine compared to the other treatments (Figure 4b). At days 7, 10 and 14, a significant reduction in cell viability was observed after treatment with chlorhexidine digluconate compared to ethanol, sodium hypochlorite, polyhexanide and the control (Figure 4b,d). In addition, after treatment with povidone iodine, a significant reduction in cell viability was observed at days 10 and 14 compared to ethanol, sodium hypochlorite, polyhexanide and the control (Figure 4c,d).

### 3.3. Common Antiseptics for Clinical Use Reduce Cell Growth and Proliferation in Skin Cell Lines

#### 3.3.1. Chlorhexidine Digluconate and Povidone Iodine Significantly Affect HaCaT Cells Growth Compared to the Other Treatments

**Figure 5:**
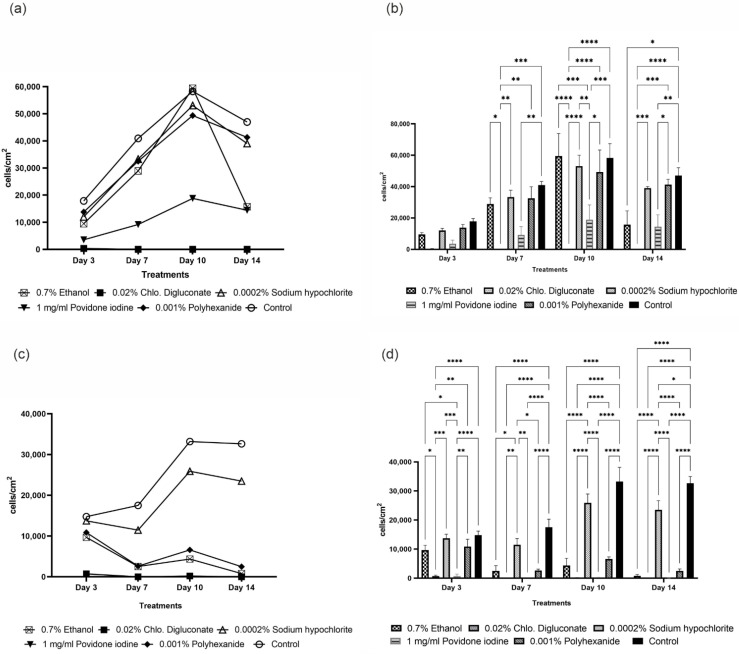
Graphical representation of cell density in HaCaT cells (**a**,**b**) and HFs (**c**,**d**) after each treatment and the control. (**a**) Line graph of cell density in HaCaT cells after each treatment and the control at days 3, 7, 10 and 14. (**b**) Bar graph with statistical analysis of cell density in HaCaT cells after antiseptic treatment and the control at days 3, 7, 10 and 14. (**c**) Line graph of cell density in HFs after each treatment and the control at days 3, 7, 10 and 14. (**d**) Bar graph with statistical analysis of cell density in HFs after antiseptic treatment and the control at days 3, 7, 10 and 14. * *p* ≤ 0.05; ** *p* ≤ 0.01; *** *p* ≤ 0.001; **** *p* ≤ 0.0001. *n* = 3. *p* values ≤ 0.05 were considered statistically significant.

In HaCaT cells, no significant differences in the number of live cells/cm^2^ were observed compared to the control and between the different treatments at day 3. On day 7, a significant reduction in cell density was observed after treatment with chlorhexidine digluconate compared to ethanol, sodium hypochlorite, polyhexanide and the control, and after treatment with povidone iodine compared to the control. On day 10, a significant decrease in cell density was observed after chlorhexidine digluconate and povidone iodine treatments with respect to ethanol, sodium hypochlorite, polyhexanide and the control. At day 14, treatment with ethanol produced a significant reduction in cell density compared to the control. Likewise, on day 14, a significant reduction in cell density was observed after treatment with chlorhexidine digluconate compared to treatments with sodium hypochlorite, polyhexanide and the control. Finally, on day 14, treatment with povidone iodine caused a significant reduction in cell density compared to treatment with polyhexanide and the control (Figure 5a,b).

#### 3.3.2. Chlorhexidine Digluconate and Povidone Iodine and Ethanol Had Greater Impact on HF Proliferation Compared to the Other Treatments

In HFs, significant differences were observed in the number of live cells/cm^2^ according to the treatment applied and compared to the control. At day 3, cell density was significantly reduced after chlorhexidine digluconate and povidone iodine treatments compared to ethanol, sodium hypochlorite, polyhexanide and the control. At day 7, treatment with ethanol significantly reduced cell density compared to sodium hypochlorite and the control. Furthermore, at day 7, treatment with chlorhexidine digluconate and povidone iodine resulted in significantly lower cell density than cells treated with sodium hypochlorite and the control. Finally, at day 7, treatment with polyhexanide significantly reduced cell density compared to the control and sodium hypochlorite. On days 10 and 14, after treatment with ethanol, chlorhexidine digluconate, povidone iodine and polyhexanide, a great impact on the number of live cells/cm^2^ was observed, a significant reduction in cell density was found compared to sodium hypochlorite and the control. Finally, at day 14, a significant reduction in cell density was observed after treatment with sodium hypochlorite compared to the control (Figure 5c,d).

### 3.4. Common Antiseptics for Clinical Use Affect Cell Migration of Skin Cell Lines

#### 3.4.1. Chlorhexidine Digluconate Inhibits Cell Migration Capacity in HaCaT Cells Compared to Other Antiseptic Treatments

**Figure 6:**
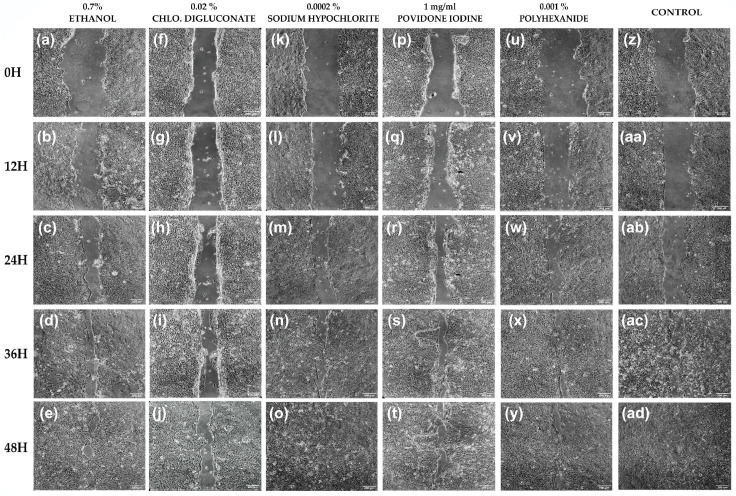
Wound closure assay on HaCaT cells (**a**–**e**) HaCaT cells after ethanol (0.7%) treatment at 0, 12, 24, 36 and 48 h after scratching, respectively. (**f**–**j**) HaCaT cells after chlorhexidine digluconate (0.02%) treatment at 0, 12, 24, 36 and 48 h after scratching, respectively. (**k**–**o**) HaCaT cells after sodium hypochlorite (0.0002%) treatment at 0, 12, 24, 36 and 48 h after scratching, respectively. (**p**–**t**) HaCaT cells after povidone iodine (1 mg/mL) treatment at 0, 12, 24, 36 and 48 h after scratching, respectively. (**u**–**y**) HaCaT cells after polyhexanide (0.001%) treatment at 0, 12, 24, 36 and 48 h after scratching, respectively. (**z**,**aa**–**ad**) Control at 0, 12, 24, 36 and 48 h after scratching, respectively. *n* = 3.

Cell migration in HaCaT cells treated with chlorhexidine digluconate was inhibited and wound closure did not occur compared to the control which closed at 48 h. With the other treatments, wound closure could be observed at 48 h and there was no significant difference between these treatments and the control (Figure 6).

**Figure 7:**
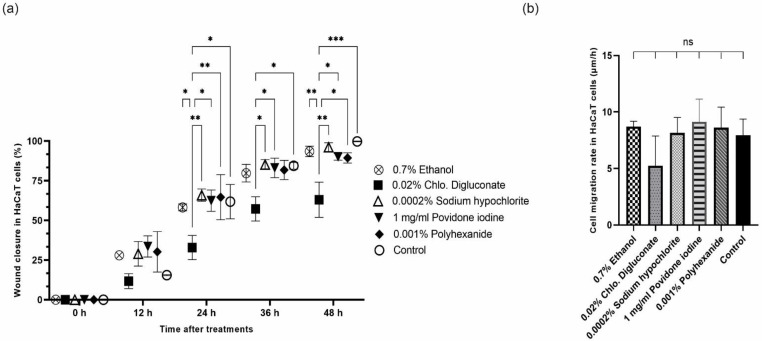
(**a**) Percentage of wound closure ± SEM for each treatment and the control in HaCaT cells at: 0, 12, 24, 36 and 48 h. (**b**) Bar graph of average cell migration rate (µm/h) after each treatment and the control in HaCaT cells. ns: no significant differences, * *p* ≤ 0.05; ** *p* ≤ 0.01; *** *p* ≤ 0.001; *n* = 3. *p* values ≤ 0.05 were considered statistically significant.

The percentage of wound closure was calculated 0, 12, 24, 36 and 48 h after applying each treatment. Between 0 and 12 h there were no significant differences in the percentage of wound closure between any treatment and the control. At 24 h, a significant inhibition of cell migration was observed after treatment with chlorhexidine digluconate compared to ethanol, sodium hypochlorite, povidone iodine, polyhexanide and the control. At 36 h, significant inhibition of wound closure was also observed after treatment with chlorhexidine digluconate compared to treatments with sodium hypochlorite, povidone iodine and the control. Finally, at 48 h, significant inhibition in wound closure was observed for cells treated with chlorhexidine digluconate compared to ethanol, sodium hypochlorite, povidone iodine, polyhexanide and the control (Figure 7a and Table S3).

In addition, a reduction in the cell migration rate was observed after treatment with chlorhexidine digluconate, however, no significant differences were observed between the different treatments and the control (Figure 7b and Table S4).

#### 3.4.2. Povidone Iodine Inhibits Cell Migration Capacity in HFs Compared to Other Antiseptics

**Figure 8:**
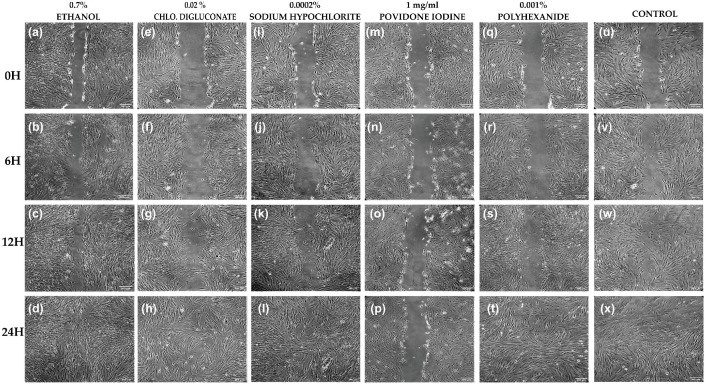
Wound closure assay on HFs. (**a**–**d**) HFs after ethanol (0.7 %) treatment at 0, 6, 12, and 24 h after scratching, respectively. (**e**–**h**) HFs after chlorhexidine digluconate (0.02%) treatment at 0, 6, 12, and 24 h after scratching, respectively. (**i**–**l**) HFs after sodium hypochlorite (0.0002%) treatment at 0, 6, 12, and 24 h after scratching, respectively. (**m**–**p**) HFs after povidone iodine (1 mg/mL) treatment at 0, 6, 12, and 24 h after scratching, respectively. (**q**–**t**) HFs after polyhexanide (0.001%) treatment at 0, 6, 12, and 24 h after scratching, respectively. (**u**–**x**) Control at 0, 6, 12, and 24 h after scratching, respectively. *n* = 3.

Cell migration was inhibited in HFs treated with povidone iodine and wound closure did not occur compared to the control which closed at 24 h. Regarding the other treatments, wound closure could be observed at 24 h and there was no significant difference between these treatments and the control (Figure 8).

**Figure 9:**
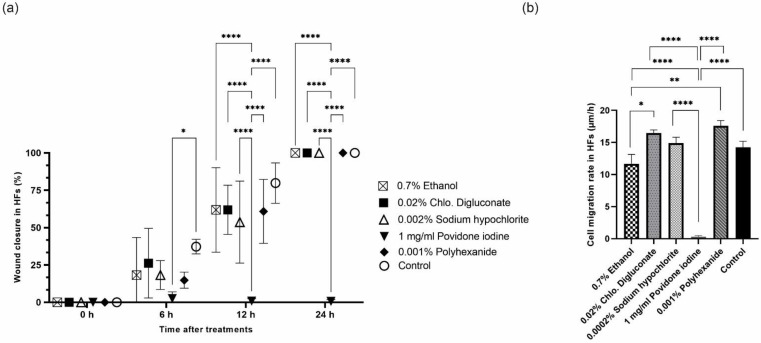
(**a**) Percentage of wound closure ± SEM for each treatment and control in HFs at: 0, 12, 24 and 36 h. (**b**) Bar graph of average cell migration rate (µm/h) after each treatment and control in HFs. ns: no significant differences, * *p* ≤ 0.05; ** *p* ≤ 0.01; **** *p* ≤ 0.0001; *n* = 3. *p* values ≤ 0.05 were considered statistically significant.

The percentage of wound closure after scraping was calculated 0, 6, 12 and 24 h after the application of each treatment. At 6 h, a significant inhibition of cell migration was observed in fibroblasts treated with povidone iodine compared to the control. At 12 and 24 h, the inhibition of wound closure after treatment with povidone iodine was significant compared to treatments with ethanol, chlorhexidine digluconate, sodium hypochlorite, polyhexanide and the control (Figure 9a and Table S5).

Therefore, 24 h after treatment, all wounds made by the scratch test had closed similarly to the control, except for fibroblasts treated with povidone iodine, which had a great impact on the migration capacity of this cell type preventing wound closure.

In addition, a significant reduction in the cell migration ratio was observed after treatment with povidone iodine compared to the other treatments and the control. The cell migration speed was also significantly reduced after treatment with ethanol compared to treatments with chlorhexidine digluconate and polyhexanide (Figure 9b and Table S6).

## 4. Discussion

Antiseptic treatments are crucial for preventing infections during epithelial regeneration in the wound healing process [14]. Current therapies in major burn patients usually include epidermal substitutions with cultured epithelial autografts and allografts, which have shown good results, but the main disadvantage is the time required to obtain them due to the long culture time [15]. New strategies are emerging as alternatives. Among these new therapies are devices based on suspensions of epithelial cells (fibroblasts and keratinocytes among others) obtained by biopsies and without the need for cell culture to promote the re-epithelialization process of the damaged area [15,16]. Therefore, due to the emergence of new therapies it is interesting to evaluate the impact of the most commonly used antiseptics in the clinic on skin cell lines, such as fibroblasts and keratinocytes. Some studies make evident the inherent cytotoxicity of antiseptics in vitro, while in vivo or clinical results seem to be controversial.

According to our results, povidone iodine, chlorhexidine digluconate and ethanol are the most cytotoxic antiseptics in skin cell lines. Chlorhexidine digluconate and ethanol reduced cell viability to 0% after 14 days of treatment in HaCaT cells. On the other hand, the viability of HFs was also reduced to 0% from day 7 after treatment with povidone iodine and from day 10 after treatment with chlorhexidine digluconate. In contrast, treatments with sodium hypochlorite did not significantly affect cell viability which remained similar to the untreated control in both cell types. In addition, in the wound closure assay, the cytotoxicity of chlorhexidine digluconate and povidone iodine could be observed, since after treatments with these antiseptics, the cell migration capacity of HaCaT cells and HFs, respectively, was inhibited, preventing wound closure compared to other treatments and the control which closed at 48 h in HaCaT cells and at 24 h in the case of HFs.

Povidone iodine is a common clinical antiseptic, effective against a wide variety of microorganisms [14,17]. It is used to treat ulcers, open wounds, burns and even on healthy skin in preparation for surgery. However, despite its germicidal power, previous studies have shown that high concentrations of povidone iodine are toxic to proliferating cells and can slow down the healing process [14]. Moreover, according to previous in vitro studies, even low concentrations can have cytotoxic effects and cause an attenuation of cell proliferation in fibroblasts, as well as in osteoblasts and myoblasts [18]. For example, Hajská et al. evaluated the potential toxic effect of 16 antiseptic treatments on murine and human dermal fibroblasts [19]. Interestingly, 24 h-treatment with povidone iodine at a concentration of 100 mg/mL was categorized as a semi-toxic agent. In this line and in agreement with the results of our study, Thomas et al. showed that povidone iodine reduces both migration and proliferation of fibroblasts in a dose-dependent fashion [20]. Furthermore, Hirsch et al. evaluated the cytotoxic effect of five min-application of different antiseptic treatments and concluded that povidone-iodine-based antiseptics showed the lowest bactericidal potential and the strongest cytotoxicity against human skin cells [21].

Our preliminary test results showed that concentrations above 1% caused total elimination of cell growth. Even low concentrations of povidone iodine produced a significant inhibition of cell viability and migration capacity in HFs that could be observed in both viability and wound closure assays. Therefore, the cytotoxic effect of povidone iodine on fibroblasts, a critical cell type for wound healing, was confirmed.

There is some controversy in the literature about the possible cytotoxic mechanisms which may be causing this inhibition of HF viability and migration capacity. For instance, Chou et al. [22] treated primary human corneal fibroblasts and a human corneal epithelial cell line with 0.1–5% of povidone-iodine for one minute and found that the mitochondrial dehydrogenase and intracellular esterase activities as well as cell membrane integrity were abolished by the treatment. They stated that the changes in membrane characteristics caused cells to become resistant to sodium dodecyl sulfate (SDS) lysis and cells did not respond to external stimuli, thus concluding that povidone iodine at 0.1% or higher causes immediate cell death through fixation rather than apoptosis or necrosis. This cytotoxic mechanism is also reported by Lee et al. [23] who support this theory that cellular fixation is the primary mechanism of cell death from povidone iodine, rather than apoptosis or necrosis. Moreover, they showed how the cytotoxic effect of this treatment is maximal at 30 min exposure to 1% povidone-iodine. However, Sato et al. [24] reported that povidone-iodine treatment induced apoptosis and necrosis in HeLa cells and rat oral mucosa after one or two days of exposure. In this line, Nomura et al. [25] studied whether apoptosis or necrosis might cause cell death after povidone iodine treatment as they found that povidone induced a significant accumulation of cells in the G0/G1 phase of the cell cycle at their IC50 concentration. Nevertheless, after analyzing the induction of apoptotic signals, they found that povidone-iodine did not induce apoptosis. Therefore, there are different hypotheses about the responsible mechanisms underlying povidone cell death. Further research including molecular techniques is necessary to confirm or refute these hypotheses.

Chlorhexidine digluconate is another of the most commonly used antiseptics for the preventing infections, preparing the surgical site, burns wounds, etc. [9,26]. According to the literature, this antiseptic is applied to the skin for three minutes and it has been shown to have inherent cytotoxicity [8,27,28]. This fact has been observed in our study. Monolayer cultures of HFs and HaCaT cells treated with chlorhexidine digluconate at the concentration used in the clinic (2%) caused 100% cell death. Even when the concentration was reduced to 0.02%, cell viability was significantly reduced in both cell types. Furthermore, the a three-minute application of chlorhexidine digluconate inhibited cell migration capacity in HaCaT cells. Therefore, the inherent cytotoxicity of these antiseptics can be confirmed.

One possible mechanism underlying this cytotoxicity reported in the literature is oxidative stress. In this way, Zhang et al. [29] reported that chlorhexidine induced reactive oxygen species (ROS) production and an upregulated expression of Nrf2, pNrf2, and HO-1 in HaCaT cells. These proteins are involved in maintaining of the intracellular oxidation-reduction homeostasis. Other authors supported the theory that chlorhexidine alters the cell cycle. Coelho et al. [30] evaluated the cell cycle of chlorhexidine-exposed human gingival fibroblasts and revealed a decrease in the number of cells in G0/G1 and an increase in the number of cells in S phase in a concentration dependent manner. This is supported by Verma et al., who found that after 1% chlorhexidine treatment, the majority of this type of cells were found to be in G0/G1 phase with very few cells in the S phase and G2/M phase of the cell cycle [31]. Finally, another reported mechanism of cell damage is a reduction in metabolic activity [30]. In our study, the mechanisms responsible for the cell viability and migration reduction were not evaluated. Therefore, further research is necessary to provide additional information in this regard.

Sodium hypochlorite is an effective antiseptic against bacteria, fungi and viruses, indicated for the care of wounds such as ulcers or burns [32]. Previous studies have shown that the viability of HFs can be reduced by up to 30% after applying sodium hypochlorite at high concentrations or undiluted [32]. In our preliminary study, different concentrations were tested, from undiluted antiseptic, which did affect cell viability, to 1% of the stock solution. Analysis of the 1% dilution of the sodium hypochlorite stock solution showed that this antiseptic does not affect cell viability in skin cell lines compared to the other treatments. In this study, a three-minute application was established according to clinical instructions. However, application time and antiseptic concentration are variable parameters in the literature. For instance, Ortega-Peña et al. demonstrated that sodium hypochlorite at 0.057% showed a recovery trend of the fibroblast population after 24 hours of treatment [33]. Accordingly, sodium hypochlorite could be selected as the least aggressive for this cell type compared to the other antiseptics tested.

The new therapies cited above for wound healing mention strategies based on cell suspensions, so studying the cytotoxic effect directly on skin cell lines may provide useful information about the clinical use of these antiseptics after treatments with these new therapies. In addition to advanced therapies based on skin cell lines, other cell sources can be used to stimulate wound healing and regeneration. The application of human perinatal cells is a promising strategy for wound treatment since they have anti-inflammatory, immunomodulatory, anti-cancer, anti-fibrotic, anti-apoptotic, and anti-oxidant effects [34]. These cells, which include human mesenchymal stem cells (hMSCs) derived from the umbilical cord (hUC-MSCs), placenta (hPMSCs) and amniotic membrane (hAMSCs) among others, have shown promising results in preclinical and clinical studies on cutaneous wound healing [34,35,36]. Therefore, these advanced therapies can benefit from the insight of this research since studying the impact of common clinical antiseptics not only in skin cell lines but also on other sources of cells such as MSCs, would provide useful information about wound care protocols after administering these therapies.

However, a limitation of this in vitro study is that it evaluates the cytotoxic effect of antiseptics on cells cultured in monolayer. This system is not representative of a well-perfused human wound. In fact, human tissue has a higher tolerance for external influences, including antiseptics, than cultured human cells [28]. Surgical wounds are a well-vascularized environment, comprised of multiple cell types and soft-tissue layers, and may have a higher tolerance for antiseptic solutions than in vitro tissue cultures [37]. Therefore, in vitro culture of skin cell lines may not accurately represent the impact on a biological system, where this cytotoxicity would be less pronounced. In this sense, research on how antiseptic treatments affect wound healing in vivo showed a different impact compared to that observed in vitro. For example, Wang et al. found that povidone iodine treatment enhanced wound healing through increased expression of transforming growth factor beta, neovascularization and re-epithelialization in a rodent model of acute skin wounds [38] and this treatment also increased the healing rate of human chronic leg ulcers [39].

To conclude, further research including in vivo studies or combinations of cytotoxicity assays with germicidal activity assays at low concentrations of antiseptics, is still required to determine the concentration and application time that do not affect cellular integrity and remain effective against microorganisms, in order to better characterize the clinical significance of the results obtained in vitro.

## Data Availability

Data is contained within the article.

## Supplementary Materials

The following supporting information can be downloaded at: https://www.mdpi.com/article/10.3390/cells11091395/s1, Figure S1: LIVE/DEAD^®^ images of HaCaT cells after ethanol treatment and control at days 3, 7, 10 and 14; Figure S2: LIVE/DEAD^®^ images of HaCaT cells after chlorhexidine digluconate treatment and control at days 3, 7, 10 and 14; Figure S3: LIVE/DEAD^®^ images of HaCaT cells after sodium hypochlorite treatment and control at days 3, 7, 10 and 14; Figure S4: LIVE/DEAD^®^ images of HaCaT cells after polyhexanide treatment and control at days 3, 7, 10 and 14; Figure S5: LIVE/DEAD^®^ images of HaCaT cells after povidone iodine treatment and control at days 3, 7, 10 and 14; Figure S6: LIVE/DEAD^®^ images of HFs after ethanol treatment and control at days 3, 7, 10 and 14; Figure S7: LIVE/DEAD^®^ images of FHs after chlorhexidine digluconate treatments and control at days 3, 7, 10 and 14; Figure S8: LIVE/DEAD^®^ images of HFs after sodium hypochlorite treatments and control at days 3, 7, 10 and 14; Figure S9: LIVE/DEAD^®^ images of HFs after sodium hypochlorite treatments and control at days 3, 7, 10 and 14; Figure S10: LIVE/DEAD^®^ images of HFs after povidone iodine treatments and control at days 3, 7, 10 and 14; Table S1: Mean cell viability percentage ± SEM for each treatment and control in HaCaT cells at days: 3, 7, 10 and 14; *n* = 3; Table S2: Mean cell viability percentage ± SEM for each treatment and control in HFs at days: 3, 7, 10 and 14; *n* = 3; Table S3: Mean wound closure percentage ± SEM for each treatment and control in HaCaT cells at hours; 12, 24, 36 and 48; *n* = 3; Table S4: Average cell migration rate (µm/h) ± SEM after each treatment and control in HaCaT cells. *n = 3*; Table S5: Mean wound closure percentage ± SEM for each treatment and control in HF at hours; 6, 12 and 24; *n* = 3; Table S6: Average cell migration rate (µm/h) ± SEM after each treatment and control in HFs *n* = 3.

## Abbreviations

BASS	Bioengineered Autologous Skin Substitute
CHLO	Chlorhexidine
DFM	Dermal Fibroblast Medium
DPBS	Dulbecco’s phosphate-buffered saline
HFs	Human Fibroblasts
hMSCs	Human Mesenchymal Stem Cells
hAMSCs	Human Amniotic Membrane derived MSCs
hPMSCs	Human Placenta derived MSCs
hUC-MSCs	Human Umbilical Cord derived MSCs
ROS	Reactive Oxygen Species
SDS	Sodium Dodecyl Sulfate
SEM	Standard Error of the Mean
