# Supplementary material for: Cytotoxicity and Wound Closure Evaluation in Skin Cell Lines after Treatment with Common Antiseptics for Clinical Use
Source: Cells. 2022 Apr 20;11(9):1395. doi: 10.3390/cells11091395 (PMC9099882; doi:10.3390/cells11091395)
Supplement: Supplementary file 1 [file cells-11-01395-s001.zip › Supplementary Figures captions.pdf]

Figure S1. LIVE / DEAD® images of HaCaT cells after ethanol treatment and control at days 3, 7, 10 and 14. (a-d) HaCaT cells after ethanol (3.5 %) treatment at days 3, 7, 10 and 14, respectively. (e-h) HaCaT cells after ethanol (7 %) treatment at day 3, 7, 10 and 14, respectively. (i-l) HaCaT cells after ethanol (35 %) treatment at days 3, 7, 10 and 14, respectively. (m-p) HaCaT cells after ethanol (70 %) treatment at days 3, 7, 10 and 14, respectively. (q-t) control at days 3, 7, 10 and 14, respectively. Dead cells are represented in red and live cells in green. Magnification 10x.

Figure S2. LIVE / DEAD® images of HaCaT cells after chlorhexidine digluconate treatment and control at days 3, 7, 10 and 14. (a-d) HaCaT cells after chlorhexidine digluconate (0.1 %) treatment at days 3, 7, 10 and 14, respectively. (e-h) HaCaT cells after chlorhexidine digluconate (0.2 %) treatment at day 3, 7, 10 and 14, respectively. (i-l) HaCaT cells after chlorhexidine digluconate (1 %) treatment at days 3, 7, 10 and 14, respectively. (m-p) HaCaT cells after chlorhexidine digluconate (2 %) treatment at days 3, 7, 10 and 14, respectively. (q-t) control at days 3, 7, 10 and 14, respectively. Dead cells are represented in red and live cells in green. Magnification 10x.

Figure S3. LIVE / DEAD® images of HaCaT cells after sodium hypochlorite treatment and control at days 3, 7, 10 and 14. (a-d) HaCaT cells after sodium hypochlorite (0.001 %) treatment at days 3, 7, 10 and 14, respectively. (e-h) HaCaT cells after sodium hypochlorite (0.002 %) treatment at day 3, 7, 10 and 14, respectively. (i-l) HaCaT cells after sodium hypochlorite (0.01 %) treatment at days 3, 7, 10 and 14, respectively. (m-p) HaCaT cells after sodium hypochlorite (0.02 %) treatment at days 3, 7, 10 and 14, respectively. (q-t) control at days 3, 7, 10 and 14, respectively. Dead cells are represented in red and live cells in green. Magnification 10x.

Figure S4. LIVE / DEAD® images of HaCaT cells after polyhexanide treatment and control at days 3, 7, 10 and 14. (a-d) HaCaT cells after polyhexanide (0.005 %) treatment at days 3, 7, 10 and 14, respectively. (e-h) HaCaT cells after polyhexanide (0.01 %) treatment at day 3, 7, 10 and 14, respectively. (i-l) HaCaT cells after polyhexanide (0.05 %) treatment at days 3, 7, 10 and 14, respectively. (m-p) HaCaT cells after polyhexanide (0.1 %) treatment at days 3, 7, 10 and 14, respectively. (q-t) control at days 3, 7, 10 and 14, respectively. Dead cells are represented in red and live cells in green. Magnification 10x.

Figure S5. LIVE / DEAD® images of HaCaT cells after povidone iodine treatment and control at days 3, 7, 10 and 14. (a-d) HaCaT cells after povidone iodine (5 mg/mL) treatment at days 3, 7, 10 and 14, respectively. (e-h) HaCaT cells after povidone iodine (10 mg/mL) treatment at day 3, 7, 10 and 14, respectively. (i-l) HaCaT cells after povidone iodine (50 mg/mL) treatment at days 3, 7, 10 and 14, respectively. (m-p) HaCaT cells after povidone iodine (100 mg/mL) treatment at days 3, 7, 10 and 14, respectively. (q-t) control at days 3, 7, 10 and 14, respectively. Dead cells are represented in red and live cells in green. Magnification 10x.

Figure S6. LIVE / DEAD® images of HFs after ethanol treatment and control at days 3, 7, 10 and 14. (a-d) HFs after ethanol (7 %) treatment at days 3, 7, 10 and 14, respectively. (e-h) HFs after ethanol (35 %) treatment at day 3, 7, 10 and 14, respectively. (i-l) HFs after ethanol (70 %)

treatment at days 3, 7, 10 and 14, respectively. (m-p) control at days 3, 7, 10 and 14, respectively. Dead cells are represented in red and live cells in green. Magnification 10x.

Figure S7. LIVE / DEAD® images of HFs after chlorhexidine digluconate treatments and control at days 3, 7, 10 and 14. (a-d) HFs after chlorhexidine digluconate (0.2 %) treatment at days 3, 7, 10 and 14, respectively. (e-h) HFs after chlorhexidine digluconate (1 %) treatment at day 3, 7, 10 and 14, respectively. (i-l) HFs after chlorhexidine digluconate (2 %) treatment at days 3, 7, 10 and 14, respectively. (m-p) control at days 3, 7, 10 and 14, respectively. Dead cells are represented in red and live cells in green. Magnification 10x.

Figure S8. LIVE / DEAD® images of HFs after sodium hypochlorite treatments and control at days 3, 7, 10 and 14. (a-d) HFs after sodium hypochlorite (0.002 %) treatment at days 3, 7, 10 and 14, respectively. (e-h) HFs after sodium hypochlorite (0.01 %) treatment at day 3, 7, 10 and 14, respectively. (i-l) HFs after sodium hypochlorite (0.02 %) treatment at days 3, 7, 10 and 14, respectively. (m-p) control at days 3, 7, 10 and 14, respectively. Dead cells are represented in red and live cells in green. Magnification 10x.

Figure S9. LIVE / DEAD® images of HFs after polyhexanide treatments and control at days 3, 7, 10 and 14. (a-d) HFs after polyhexanide (0.01 %) treatment at days 3, 7, 10 and 14, respectively. (e-h) HFs after polyhexanide (0.05 %) treatment at day 3, 7, 10 and 14, respectively. (i-l) HFs after polyhexanide (0.1 %) treatment at days 3, 7, 10 and 14, respectively. (m-p) control at days 3, 7, 10 and 14, respectively. Dead cells are represented in red and live cells in green. Magnification 10x.

Figure S10. LIVE / DEAD® images of HFs after povidone iodine treatments and control at days 3, 7, 10 and 14. (a-d) HFs after povidone iodine (10 mg/mL) treatment at days 3, 7, 10 and 14, respectively. (e-h) HFs after povidone iodine (50 mg/mL) treatment at day 3, 7, 10 and 14, respectively. (i-l) HFs after povidone iodine (100 mg/mL) treatment at days 3, 7, 10 and 14, respectively. (m-p) control at days 3, 7, 10 and 14, respectively. Dead cells are represented in red and live cells in green. Magnification 10x.

Table S1. Mean cell viability percentage ± SEM for each treatment and control in HaCaT cells at days: 3, 7, 10 and 14; *n*= 3.

Table S2. Mean cell viability percentage ± SEM for each treatment and control in HFs at days: 3, 7, 10 and 14; *n*= 3.

Table S3. Mean wound closure percentage ± SEM for each treatment and control in HaCaT cells at hours; 12, 24, 36 and 48; *n*= 3.

Table S4. Average cell migration rate (µm/h) ± SEM after each treatment and control in HaCaT cells. *n*= 3.

Table S5. Mean wound closure percentage ± SEM for each treatment and control in HF at hours; 6, 12 and 24; *n*= 3.

Table S6. Average cell migration rate (µm/h) ± SEM after each treatment and control in HFs. *n*= 3.
