# Supplementary material for: Cytotoxicity and Wound Closure Evaluation in Skin Cell Lines after Treatment with Common Antiseptics for Clinical Use
Source: Cells. 2022 Apr 20;11(9):1395. doi: 10.3390/cells11091395 (PMC9099882; doi:10.3390/cells11091395)
Supplement: Supplementary file 1 [file cells-11-01395-s001.zip › Table S1.pdf]

Table S1. Mean cell viability percentage ± SEM for each treatment and control in HaCaT cells at days: 3, 7, 10 and 14; *n*= 3.

Treatments	Day 3	Day 7	Day 10	Day 14
Ethanol (0.7 %)	99.32 ± 0.61	97.18 ± 1.53	94.19 ± 4.04	0.64 ± 0.39
Chlorhexidine digluconate (0.02 %)	24.18 ± 2.46	24.76 ± 13.21	26.31 ± 19.21	0.00 ± 0
Sodium hypochlorite (0.0002 %)	99.64 ± 0.33	99.31 ± 0.46	99.39 ± 0.3	97.76 ± 0.54
Povidone iodine (1 mg/mL)	74.1 ± 20.92	60.28 ± 30.49	65.65 ± 32.84	57.07 ± 28.64
Polyhexanide (0.001 %)	99.5 ± 0.1	99.46 ± 0.26	99.95 ± 0.04	97.18 ± 1.28
Control	99.95 ± 0.03	99.29 ± 0.18	99.25 ± 0.43	97.17 ± 0.38
