# Supplementary material for: Cytotoxicity and Wound Closure Evaluation in Skin Cell Lines after Treatment with Common Antiseptics for Clinical Use
Source: Cells. 2022 Apr 20;11(9):1395. doi: 10.3390/cells11091395 (PMC9099882; doi:10.3390/cells11091395)
Supplement: Supplementary file 1 [file cells-11-01395-s001.zip › Table S2.pdf]

Table S2. Mean cell viability percentage ± SEM for each treatment and control in HFs at days: 3, 7, 10 and 14; *n*= 3.

**Treatments**	**Day 3**	**Day 7**	**Day 10**	**Day 14**
Ethanol (0.7 %)	93.33 ± 6.55	94.49 ± 2.6	77.74 ± 13	77.28 ± 9.97
Chlorhexidine digluconate (0.02 %)	57.99 ± 3.64	40.95 ± 23.02	0.00 ± 0	0.00 ± 0
Sodium hypochlorite (0.0002 %)	98.63 ± 0.47	94.92 ± 2.67	97.51 ± 0.73	98.87 ± 0.2
Povidone iodine (1 mg/mL)	38.95 ± 14.17	0.00 ± 0	0.00 ± 0	0.00 ± 0
Polyhexanide (0.001 %)	95.91 ± 3.22	96.32 ± 0.11	95.75 ± 2.27	78.59 ± 3.83
Control	98.47 ± 1.44	98.37 ± 0.89	96.52 ± 2.29	99.48 ± 0.2
