# Supplementary material for: Cytotoxicity and Wound Closure Evaluation in Skin Cell Lines after Treatment with Common Antiseptics for Clinical Use
Source: Cells. 2022 Apr 20;11(9):1395. doi: 10.3390/cells11091395 (PMC9099882; doi:10.3390/cells11091395)
Supplement: Supplementary file 1 [file cells-11-01395-s001.zip › Table S3.pdf]

Table S3. Mean wound closure percentage ± SEM for each treatment and control in HaCaT cells at hours; 12, 24, 36 and 48; *n*= 3.

Treatments	12 h	24 h	36 h	48 h
Ethanol (0.7 %)	28.03 ± 2.84	58.22 ± 2.17	79.76 ± 5.54	93.47 ± 3.26
Chlorhexidine digluconate (0.02 %)	11.71 ± 4.73	32.90 ± 7.63	57.25 ± 7.66	63.00 ± 11.11
Sodium hypochlorite (0.0002 %)	28.96 ± 7.72	65.89 ± 3.85	85.20 ± 3.03	96.10 ± 2.84
Povidone iodine (1 mg/mL)	33.56 ± 6.68	62.47 ± 6.68	83.06 ± 6.14	90.05 ± 2.07
Polyhexanide (0.001 %)	30.21 ± 12.75	64.62 ± 14.18	81.79 ± 6.06	89.36 ± 3.21
Control	15.5 ± 0.33	61.79 ± 10.80	84.45 ± 2.13	99.77 ± 0.23
