# Supplementary material for: Cytotoxicity and Wound Closure Evaluation in Skin Cell Lines after Treatment with Common Antiseptics for Clinical Use
Source: Cells. 2022 Apr 20;11(9):1395. doi: 10.3390/cells11091395 (PMC9099882; doi:10.3390/cells11091395)
Supplement: Supplementary file 1 [file cells-11-01395-s001.zip › Table S4.pdf]

Table S4. Average cell migration rate (µm/h) ± SEM after each treatment and control in HaCaT cells. *n*= 3.

Treatments	Cell migration rate in HaCaT cells (µm/h)
Ethanol (0.7 %)	8.71 ± 0.47
Chlorhexidine digluconate (0.02 %)	5.25 ± 2.63
Sodium hypochlorite (0.0002 %)	8.14 ± 1.37
Povidone iodine (1 mg/mL)	9.13 ± 2.02
Polyhexanide (0.001 %)	8.62 ± 1.82
Control	7.94 ± 1.43
