# Supplementary material for: Cytotoxicity and Wound Closure Evaluation in Skin Cell Lines after Treatment with Common Antiseptics for Clinical Use
Source: Cells. 2022 Apr 20;11(9):1395. doi: 10.3390/cells11091395 (PMC9099882; doi:10.3390/cells11091395)
Supplement: Supplementary file 1 [file cells-11-01395-s001.zip › Table S5.pdf]

Table S5. Mean wound closure percentage ± SEM for each treatment and control in HF at hours; 6, 12 and 24; *n*= 3.

Treatments	6 h	12 h	24 h
Ethanol (0.7 %)	18.22 ± 14.53	61.87 ± 16.33	100.00 ± 0.00
Chlorhexidine digluconate (0.02 %)	25.09 ± 14.53	61.92 ± 9.50	100.00 ± 0.00
Sodium hypochlorite (0.0002 %)	18.26 ± 5.58	53.66 ± 15.82	100.00 ± 0.00
Povidone iodine (1 mg/mL)	2.55 ± 2.55	0.89 ± 0.89	0.67 ± 0.67
Polyhexanide (0.001 %)	14.82 ± 3.10	60.89 ± 12.37	100.00 ± 0.00
Control	37.29 ± 2.83	79.83 ± 7.81	100.00 ± 0.00
