# Supplementary material for: Cytotoxicity and Wound Closure Evaluation in Skin Cell Lines after Treatment with Common Antiseptics for Clinical Use
Source: Cells. 2022 Apr 20;11(9):1395. doi: 10.3390/cells11091395 (PMC9099882; doi:10.3390/cells11091395)
Supplement: Supplementary file 1 [file cells-11-01395-s001.zip › Table S6.pdf]

Table S6. Average cell migration rate (µm/h) ± SEM after each treatment and control in HFs. *n*= 3.

Treatments	Cell migration rate in HFs (µm/h)
Ethanol (0.7 %)	11.69 ± 1.46
Chlorhexidine digluconate (0.02 %)	16.48 ± 0.48
Sodium hypochlorite (0.0002 %)	14.98 ± 0.9
Povidone iodine (1 mg/mL)	0.3 ± 0.2
Polyhexanide (0.001 %)	17.59 ± 0.84
Control	14.25 ± 0.95
