# Supplementary figures and images for: Cytotoxicity and Wound Closure Evaluation in Skin Cell Lines after Treatment with Common Antiseptics for Clinical Use
Source: Cells. 2022 Apr 20;11(9):1395. doi: 10.3390/cells11091395 (PMC9099882; doi:10.3390/cells11091395)

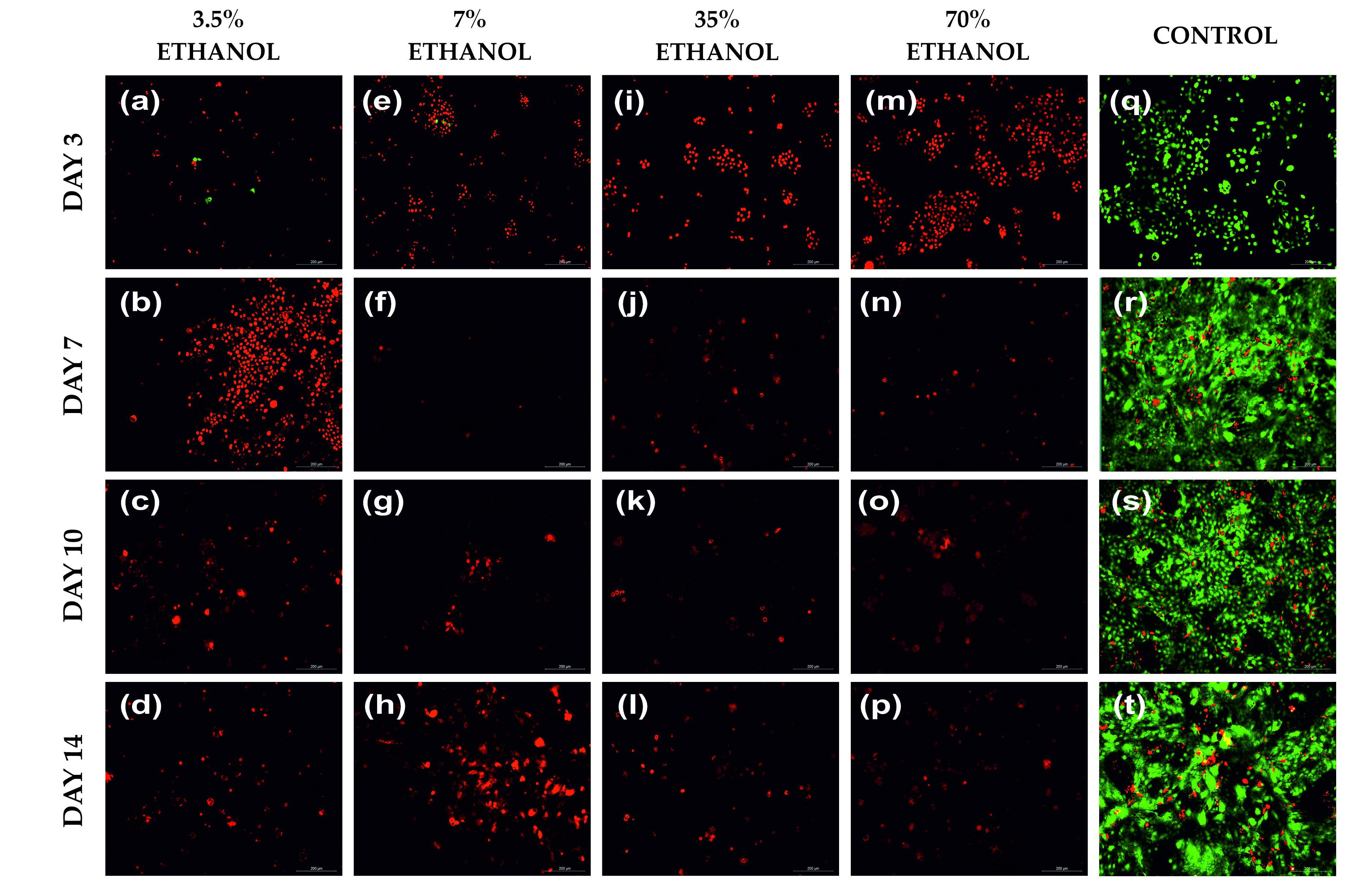

Supplement: Supplementary file 1 [file cells-11-01395-s001.zip › Figure S1.tiff]

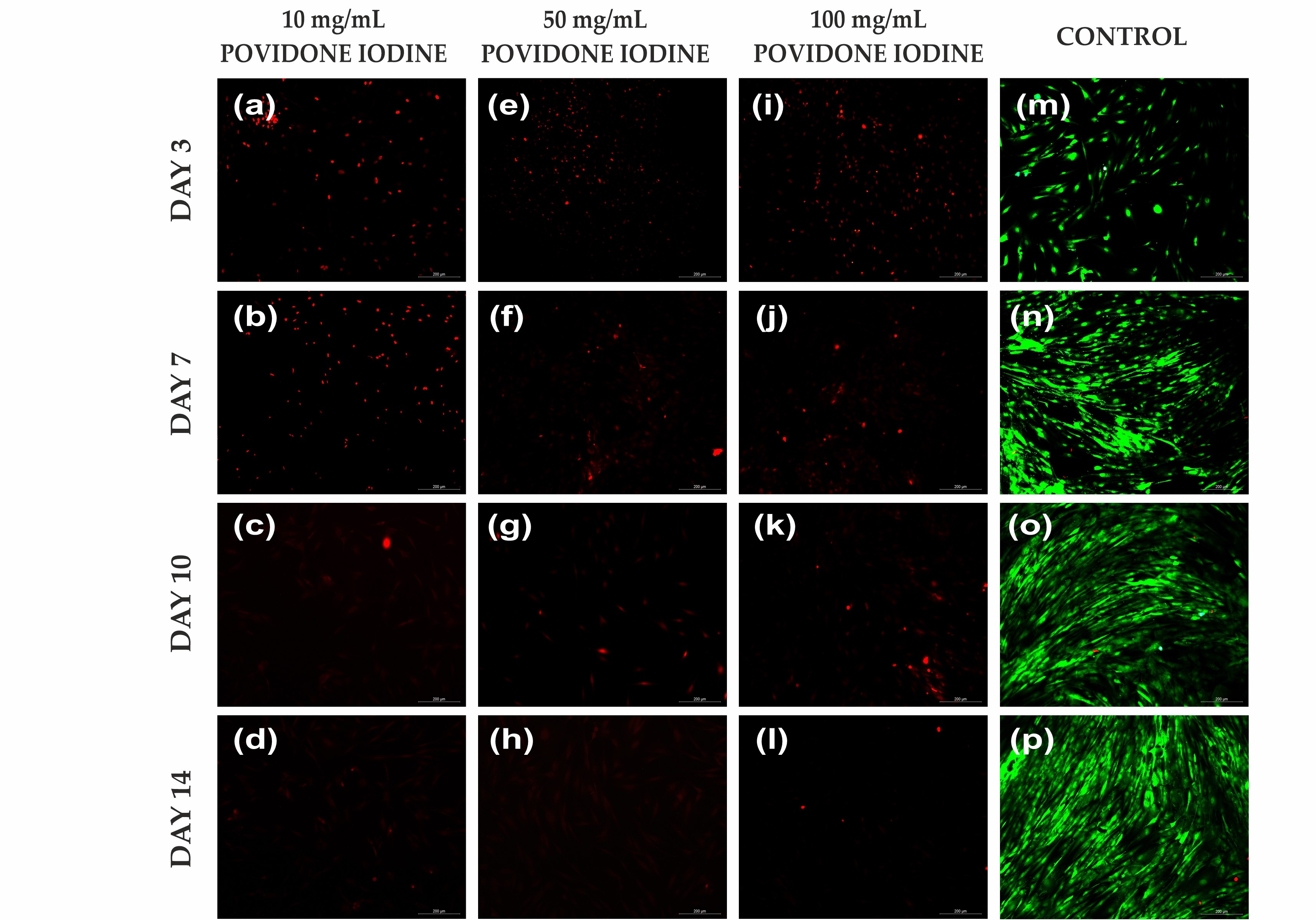

Supplement: Supplementary file 1 [file cells-11-01395-s001.zip › Figure S10.tiff]

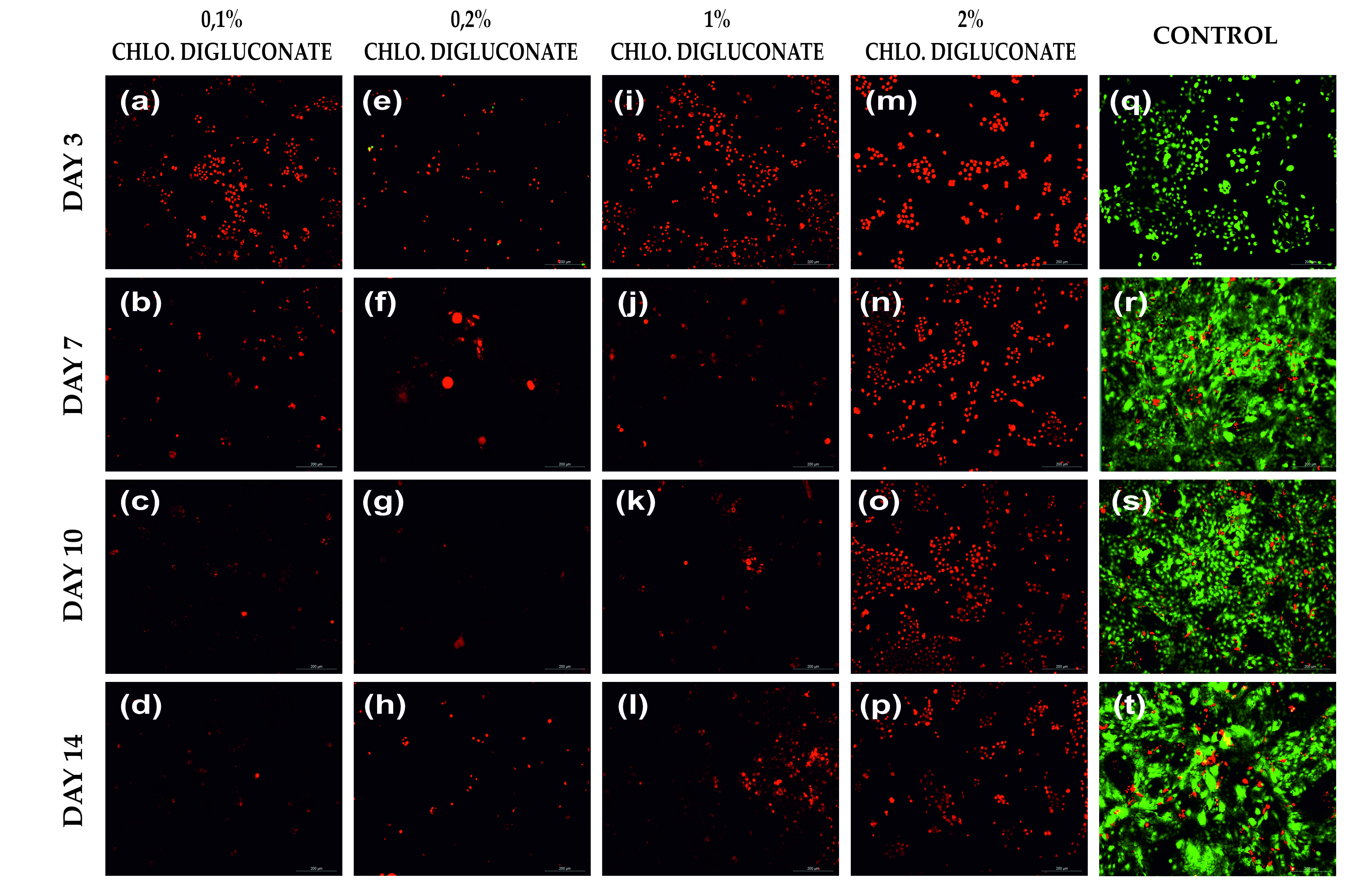

Supplement: Supplementary file 1 [file cells-11-01395-s001.zip › Figure S2.tiff]

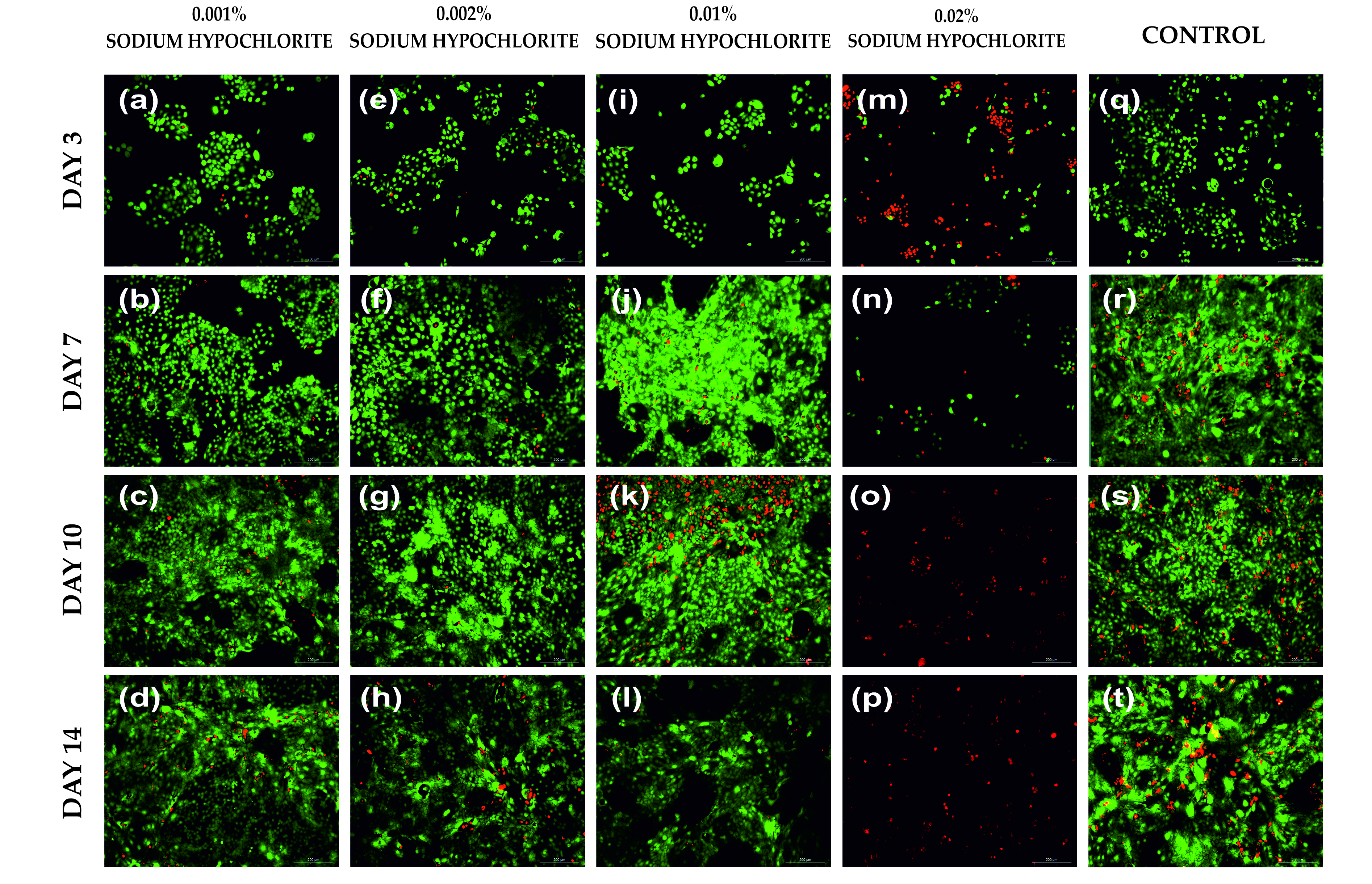

Supplement: Supplementary file 1 [file cells-11-01395-s001.zip › Figure S3.tiff]

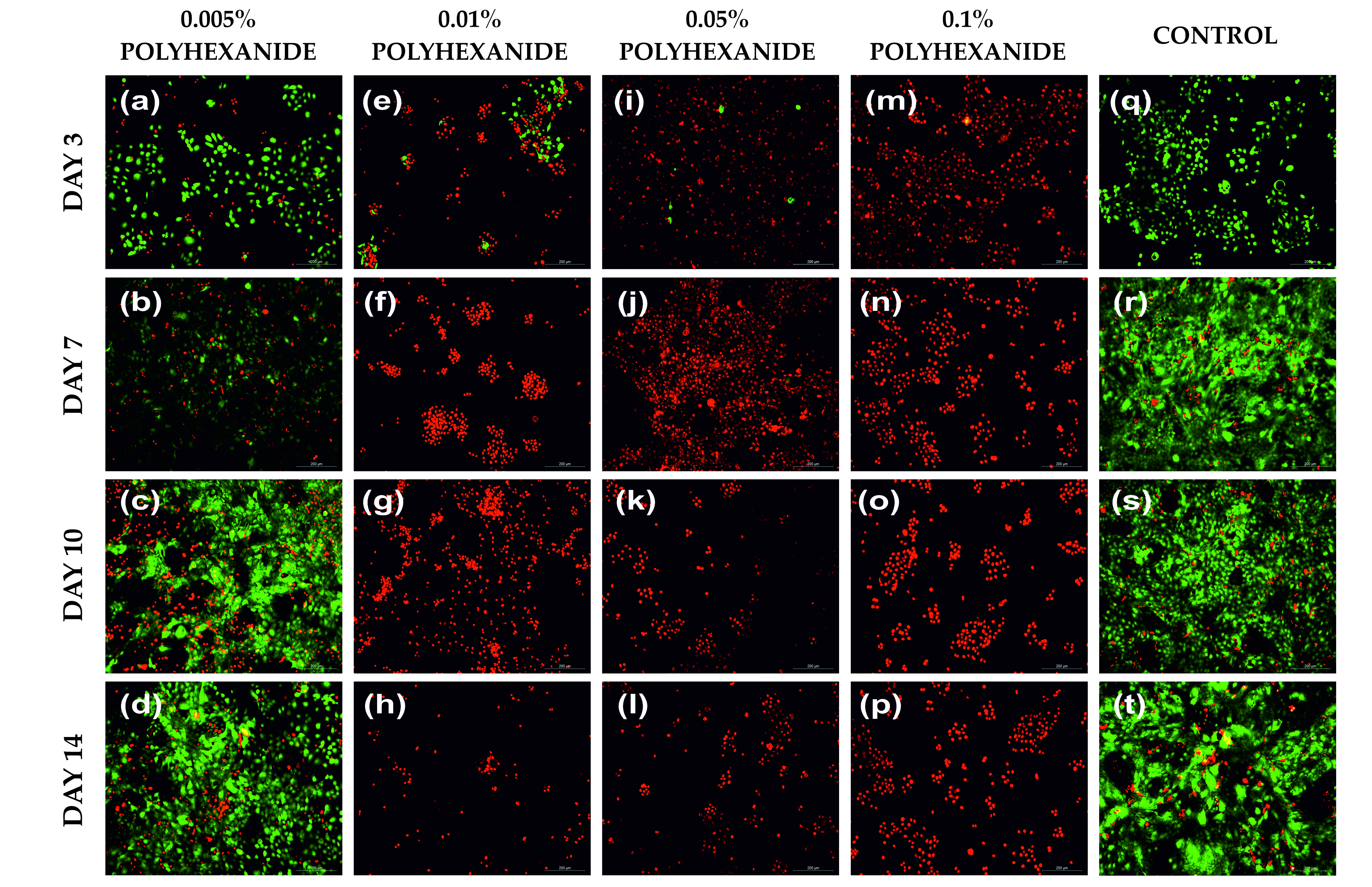

Supplement: Supplementary file 1 [file cells-11-01395-s001.zip › Figure S4.tiff]

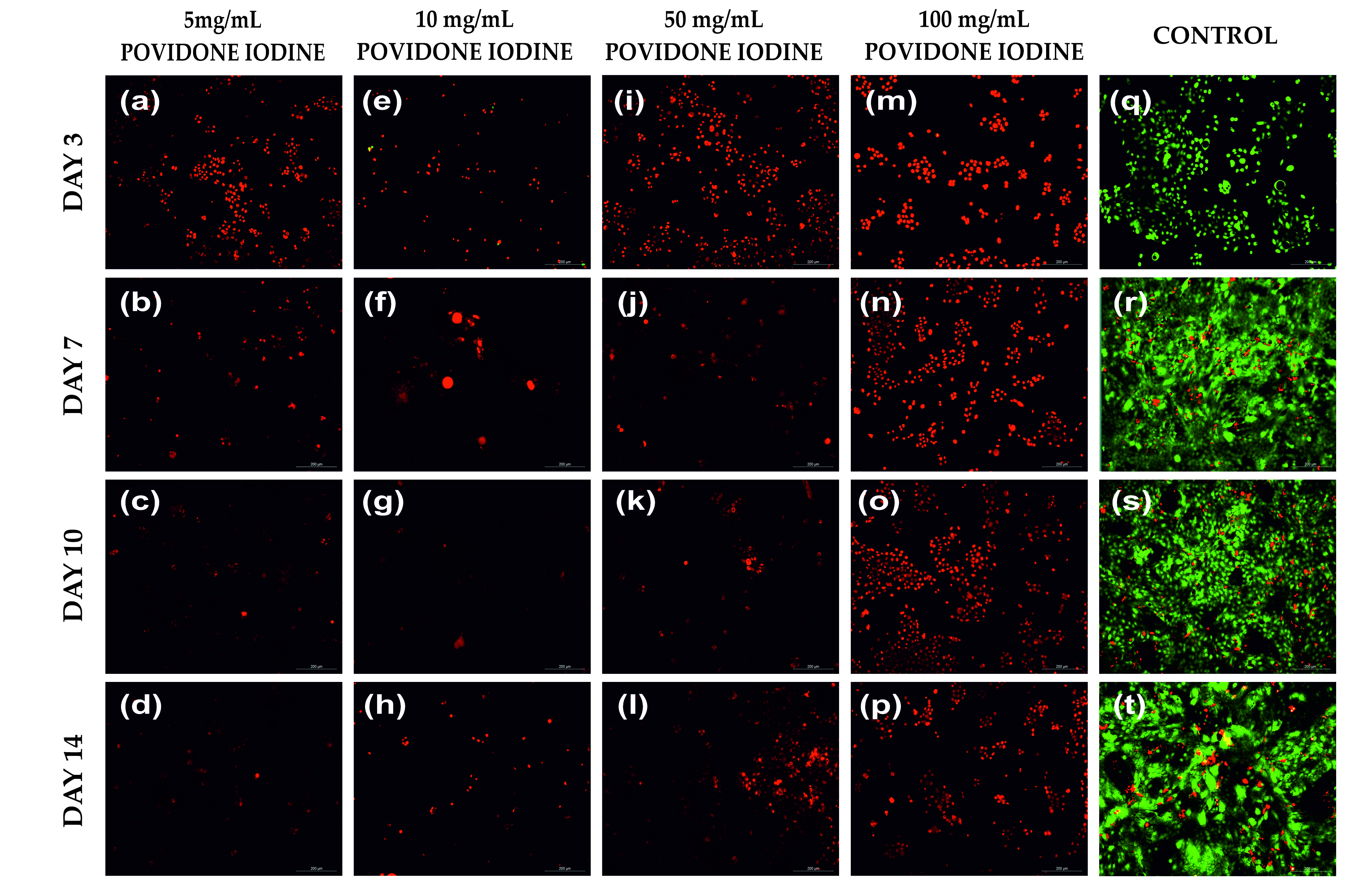

Supplement: Supplementary file 1 [file cells-11-01395-s001.zip › Figure S5.tiff]

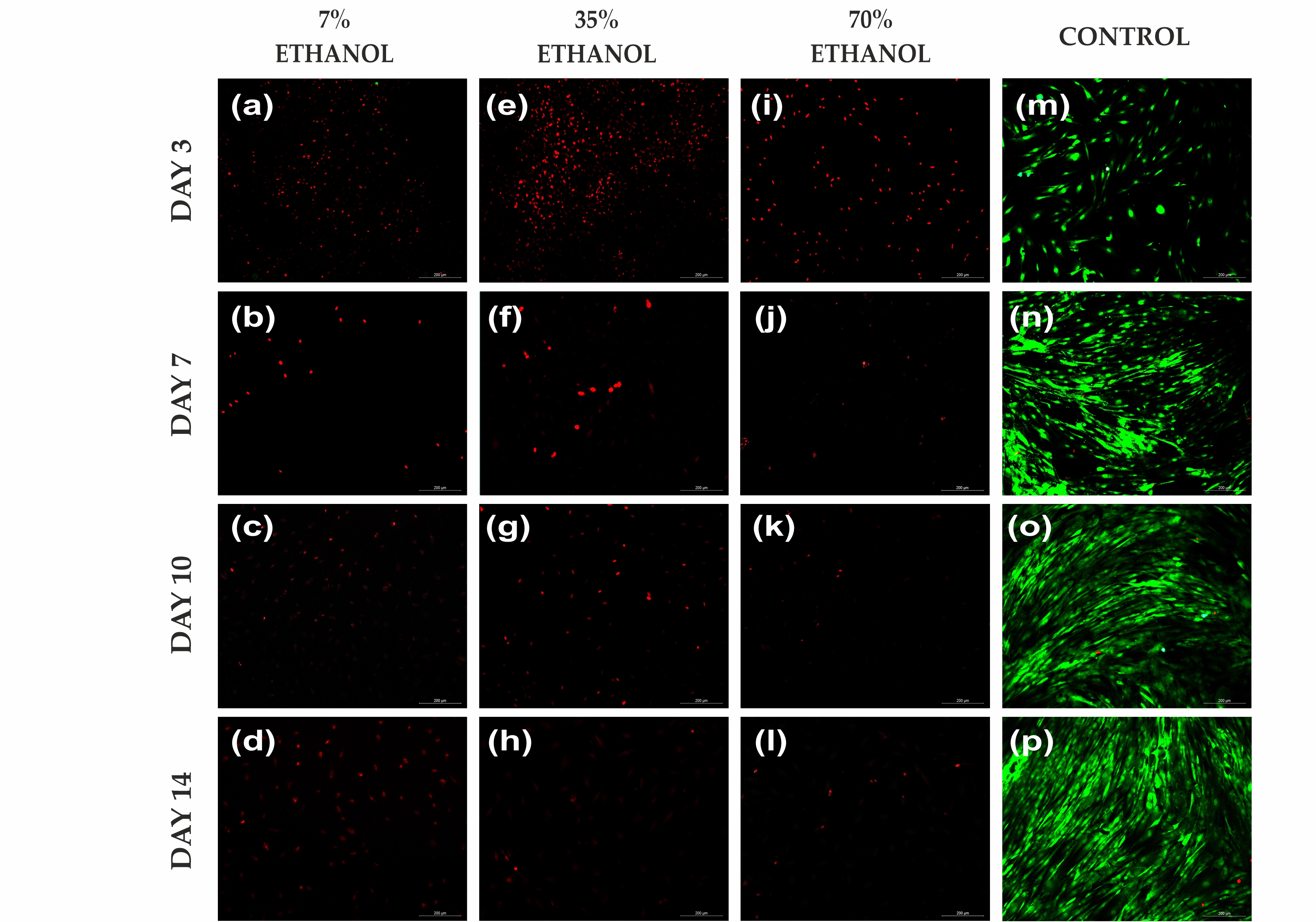

Supplement: Supplementary file 1 [file cells-11-01395-s001.zip › Figure S6.tiff]

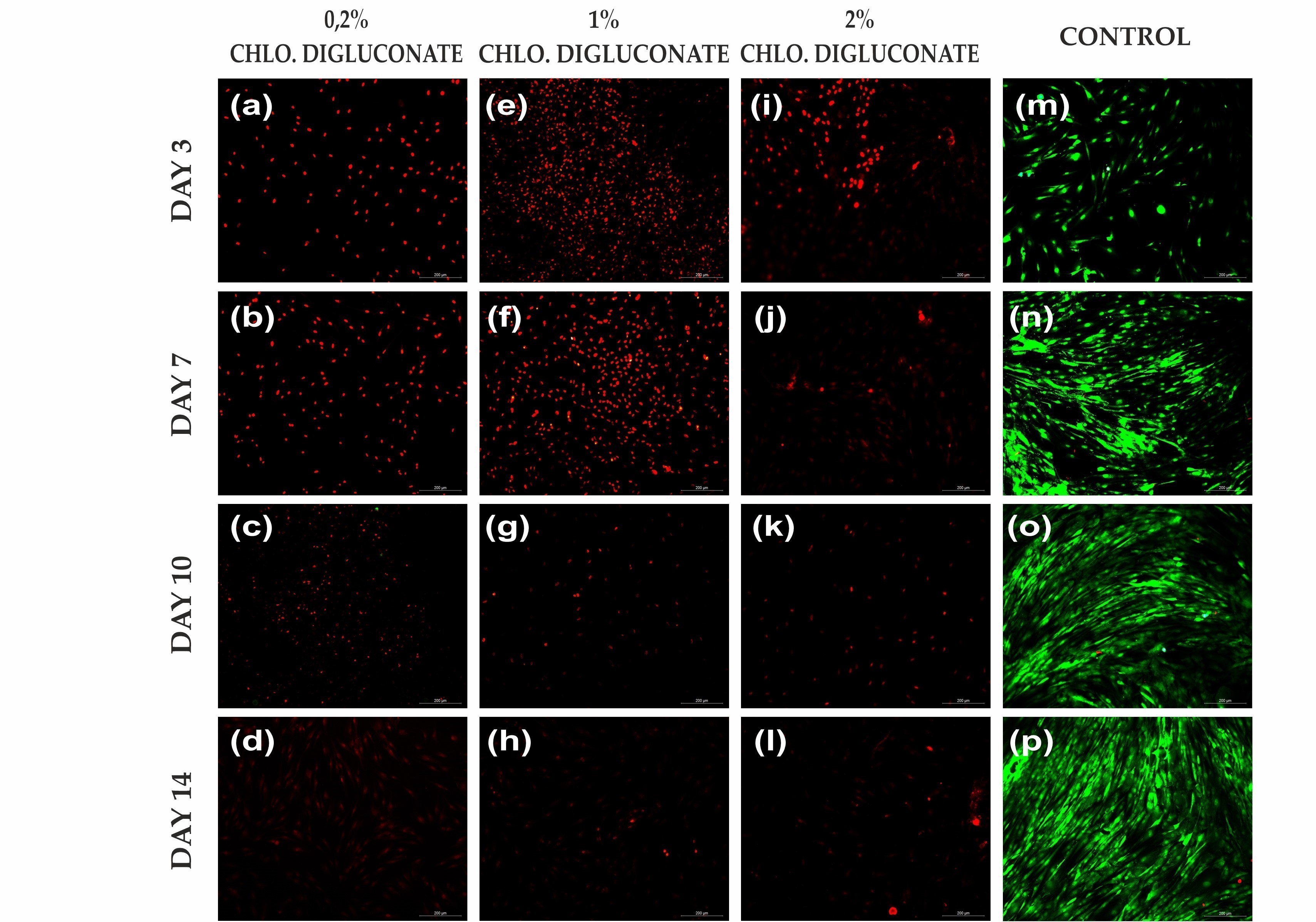

Supplement: Supplementary file 1 [file cells-11-01395-s001.zip › Figure S7.tiff]

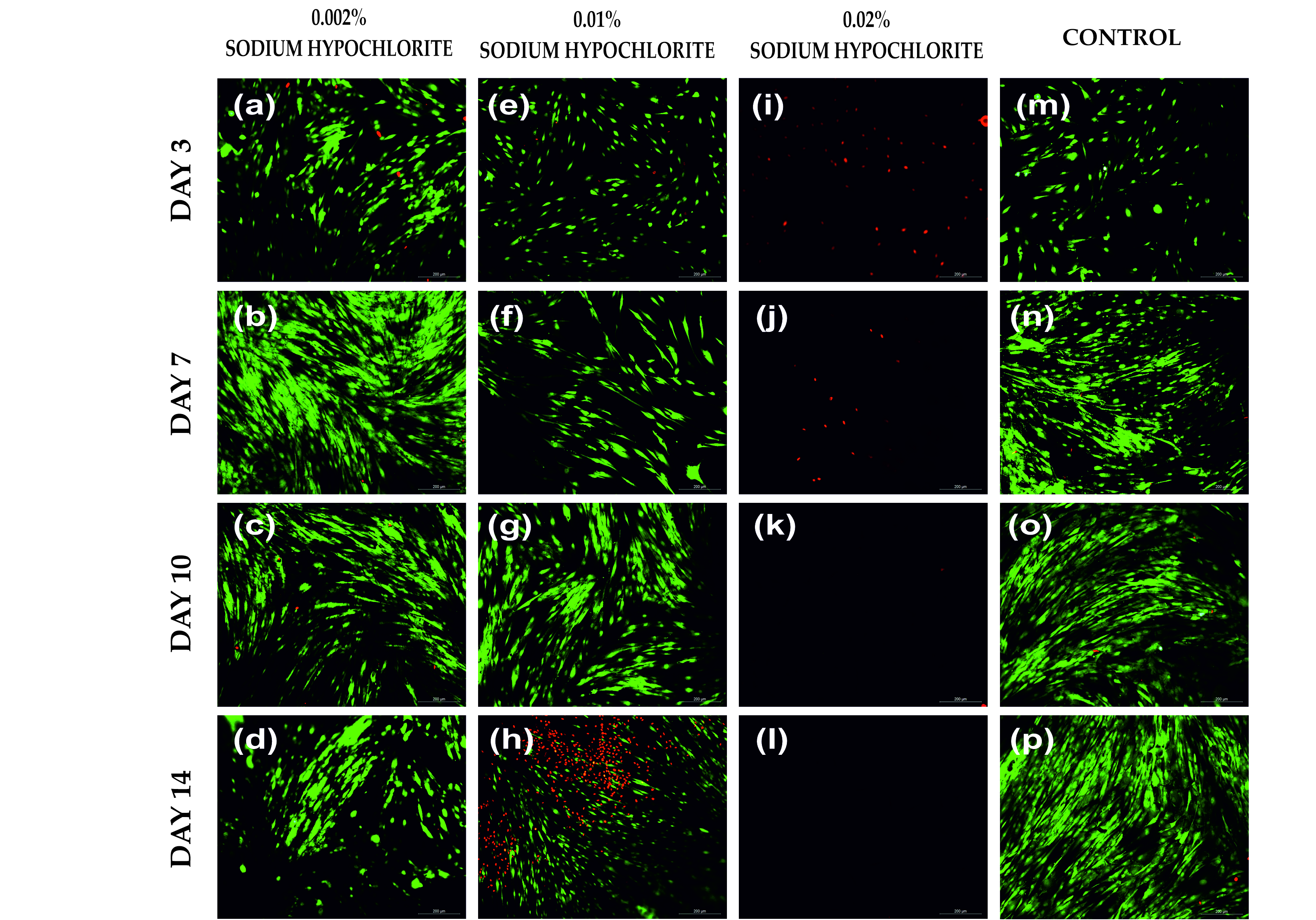

Supplement: Supplementary file 1 [file cells-11-01395-s001.zip › Figure S8.tiff]

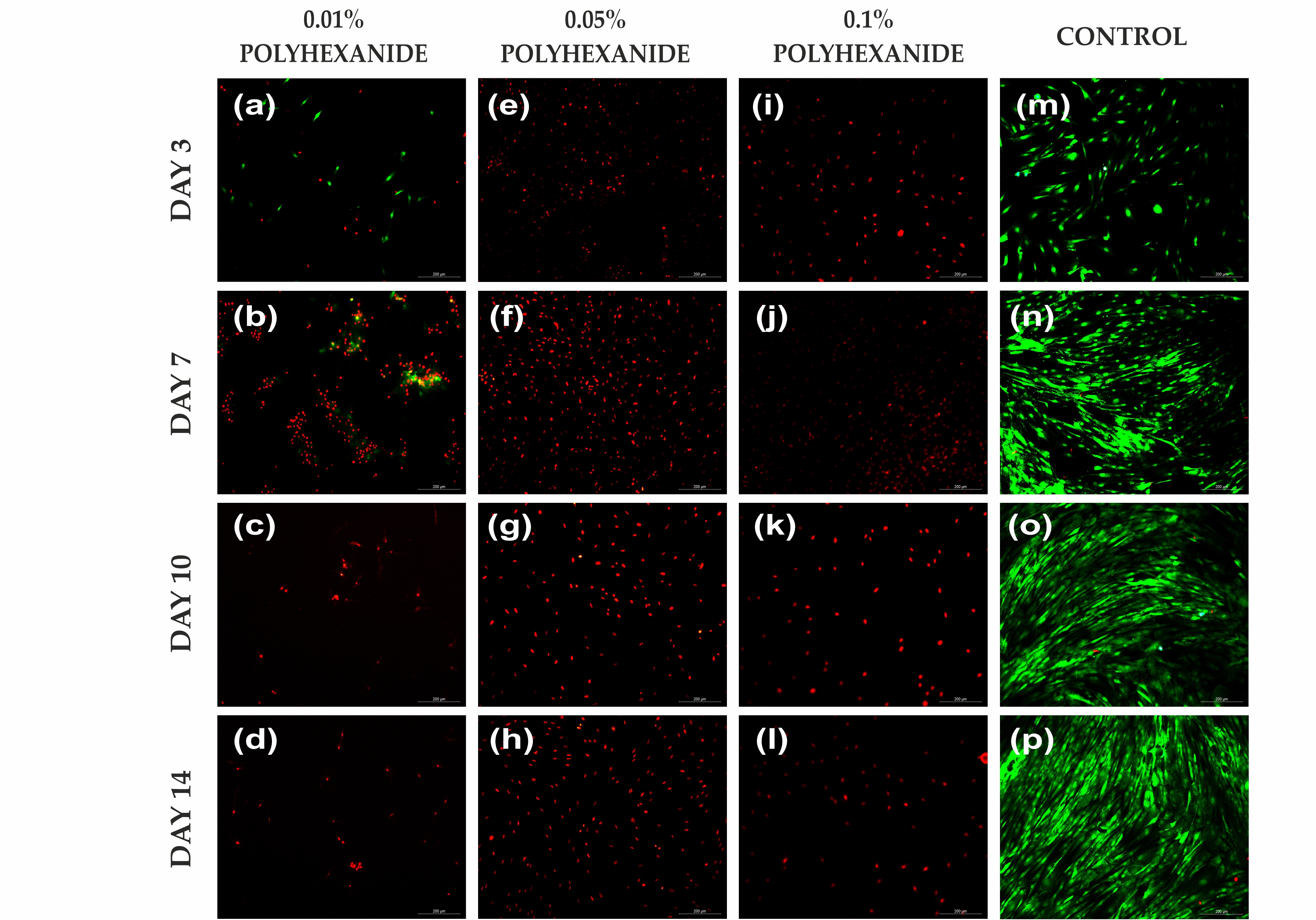

Supplement: Supplementary file 1 [file cells-11-01395-s001.zip › Figure S9.tiff]
